# Are We Looking in the Wrong Place? Implications for Behavioural-Based Pain Assessment in Rabbits (*Oryctolagus cuniculi*) and Beyond?

**DOI:** 10.1371/journal.pone.0013347

**Published:** 2011-03-15

**Authors:** Matthew C. Leach, Claire A. Coulter, Claire A. Richardson, Paul A. Flecknell

**Affiliations:** Institute of Neuroscience, Newcastle University, Newcastle upon Tyne, United Kingdom; University of Rennes 1, France

## Abstract

**Background:**

Successful observation of behaviour depends upon knowing both which behaviours to look for and focusing on the appropriate areas of the body to observe them. Behaviour based scoring systems have become increasingly widely used to assess animal pain and distress. Although studies are available demonstrating which behaviours need to be observed, there has been little attempt to assess how effectively observers apply such information when viewing an animal's behaviour.

**Methodology/Principal Findings:**

This study used historical video recordings of New Zealand white rabbits (Oryctolagus cuniculi) considered to be experiencing varying degrees of post-operative pain to assess the pattern of observation and the ability to assess pain exhibited by both experienced and inexperienced human participants (n = 151). Eye tracking equipment was used to identify how quickly, how frequently, for how long different areas of the rabbit's body were attended to by the participants. Simple visual analgoue scoring was used to assess the pain experienced in each sequence. The results demonstrate that irrespective of their experience or gender, observers focus first, more frequently and for longer on the face, compared to the abdomen, ears, back and hindquarters of the rabbit and that participants were poor at identifying rabbits in pain. Observing the back and hindquarters was correlated with ‘correct’ assessments and observing the face was correlated with ‘incorrect’ assessments.

**Conclusions:**

In conclusion, irrespective of experience and gender, observers focused on the face when using behaviour to assess pain and were unable to effectively identify rabbits in pain. Focusing on the face is unlikely to be effective when using behavioural indicators of pain since they involve other body areas. Alternatively, if animals exhibit pain-related facial expressions, then it could improve our ability to assess pain. In addition, these results have potential implications for the use of behaviour to assess how rabbits and potentially other species feel.

## Introduction

The observation and recording of behaviour is a fundamental tool in a wide range of disciplines including: ethology, psychology conversation biology, animal welfare, veterinary medicine and behavioural pharmacology. Irrespective of the reason for measuring behaviour, successful assessment depends upon knowing which behaviours to record and attending to the correct place frequently enough and for long enough to observe these behaviours. It could be argued that these two criteria are mutually inclusive; as by knowing which behaviour to record we intuitively know where to look and therefore focus our attention in the correct place at an appropriate frequency and duration. However, there is little objective evidence to support these assumptions, as there has been little or no attempt to objectively assess where and how observers focus their attention when recording animal behaviour.

Ensuring that we attend in an appropriate manner is of particular importance when observing behaviours that are subtle, infrequent, short-duration, novel or location-specific. One area where this is likely to be particularly critical is for behaviour-based pain assessment schemes that have been developed and implemented for a wide range of animal species [1]–[10]. If it is found that we do not attend to animals appropriately when observing them, this may limit our ability to assess pain in many species despite an increasing body of literature detailing which behaviours are indicative of pain [1], [11]–[13].

Automated eye-tracking has become a widely used method of assessing where people focus their attention during a range of activities, e.g. road sign recognition [14], playing chess [15], learning to drive [16], watching movies [17] and using computers [18]. Recently, Cornelissen et al. [19] have demonstrated effectiveness of this technique for tracking which areas of the human body observers focus on when judging female attractiveness. Therefore automated eye-tracking could easily and effectively be applied to the assessment of where and how people attend when observing an animal's behaviour. Eye-tracking is considered to have a number of advantages over alternative measures (e.g. self-report) as it constantly records everywhere an observer focuses their attention no matter how fleetingly. It is unaffected by whether the observer is conscious or unconscious of where they are focusing their attention. It can easily and accurately measure a wide range of parameters including the frequency and duration of observations. It is unaffected by observer preconceptions and therefore can be considered more reliable. Observer preconceptions are particular concern when dealing with an emotive subject such as animal pain. Observers may intentionally or unintentionally falsely report the pattern of their observations due to concern over failure to observe the animal appropriately [unpublished observations].

Automated eye tracking was used to investigate which areas of the body observers' focus on when attempting to assess post-surgical pain in rabbits. This included assessments of how observer gender and experience (with rabbits) influenced observation patterns. This assessment was chosen because rabbits are commonly kept as pets and used as laboratory animals. A large proportion of rabbits undergo at least one potentially painful procedure during their lifetime [20], [21] and rabbit pain is considered difficult to assess [1].

## Results

**Figure 1:**
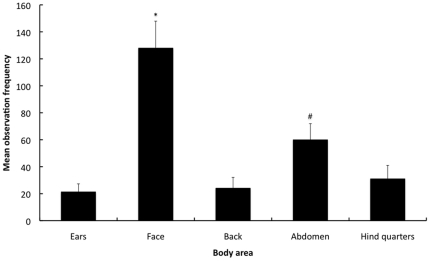
Mean observation frequencies of the body areas during the 1 min sequence. Corrected observation frequencies are presented on the y-axis (± 1SE) and areas of the body are presented on the x-axis. [* = Significantly greater observation frequency compared to all other body areas (P<0.001), # = Significantly greater observation frequency compared to the back, hind quarters & ears (P<0.001)].

**Figure 2:**
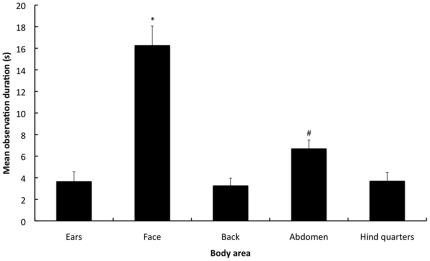
Mean observation durations of the body areas during the 1 min sequence. Corrected observation durations (seconds) are presented on the y-axis (± 1SE) and areas of the body are presented on the x-axis. [* = Significantly greater observation duration compared to all other body areas (P<0.001), # = Significantly greater observation duration compared to the back, hind quarters & ears (P<0.001)].

**Figure 3:**
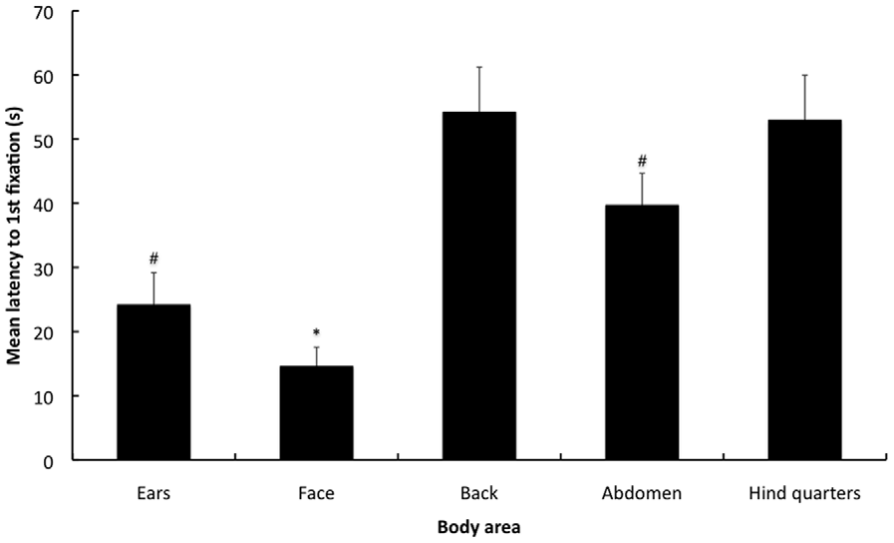
Mean latencies to 1st fixation of the body areas during the 1 min sequence. Corrected latencies to 1st fixation (seconds) are presented on the y-axis (± 1SE) and areas of the body are presented on the x-axis. [* = Significantly lower latency to 1^st^ fixation compared to all other body areas (P<0.001), # = Significantly lower latency to 1^st^ fixation compared to the back & hind quarters (P<0.001)].

The observation frequency and duration was significantly different between the various areas of the body (F1.4,163.8 = 123.0, P<0.001; F1.68,195.3 = 112.4, P<0.001, respectively) (Figures 1 & 2). The face was observed significantly more frequently and for longer compared to the abdomen, hindquarters, back and ears (P<0.001 for all comparisons). The abdomen was observed significantly more frequently and for longer compared to the back, hindquarters and ears (P<0.001 for all comparisons). There was no significant difference in the observation frequency or duration between the hindquarters, back and ears. The latency to 1st fixation was significantly different between the various areas of the body (F2.8,323.6 = 216.7, P<0.001) (Figure 3). The face had a significantly shorter latency to the 1st fixation compared to the ears, abdomen, hindquarters and back (P<0.001 for all comparisons). The ears and abdomen had a significantly shorter latencies to the 1st fixation compared to the hindquarters and back (P<0.001 for all comparisons). There were no significant differences in the latency to 1st fixation between the hindquarters and back or between the ears and abdomen.

Video sequence had a significant effect on observation frequency and duration (F2.8,319 = 16.0, P<0.001; GLM: F2.8,319 = 13.2, P<0.001, respectively) and on latency to 1st fixation on each of the body areas (GLM: F2.8,320 = 26.0, P<0.001). All body areas were observed significantly more frequently and for longer in sequence 4 (severe pain) compared to sequences 1 (normal), 2 (mild pain) and 3 (moderate pain) (P<0.001 for all comparisons). Observation frequency and duration of the body areas were not different between sequences 1, 2 and 3. Latencies to 1st fixation of all body areas were significantly longer for; sequence 1 (normal) compared to sequences 3 (moderate) and 4 (severe) (P<0.001 for both comparisons); sequence 2 compared to 3 (P<0.001), and sequence 3 compared to 4 (P<0.01).

**Figure 4:**
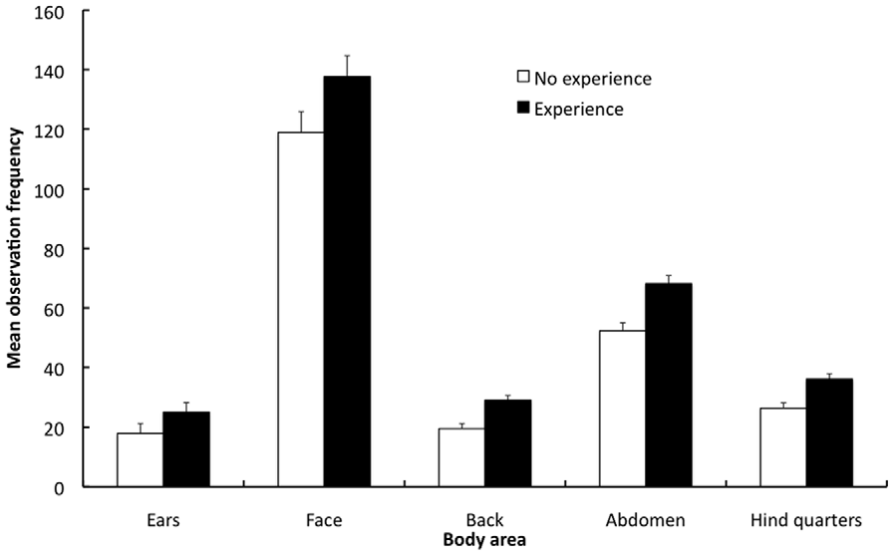
Mean observation frequencies of the body areas by observer experience during the 1 min sequence. Areas of the body are presented on the x-axis and corrected observation frequencies are presented on the y-axis (± 1SE). The open bars represent the observation frequencies of the observers with no experience of rabbits. The closed bars represent the observation frequencies of the observers with experience of rabbits.

The experience and gender of the observer did not have significant effects on observation frequency or duration or the latency to 1st fixation of any of the body areas. However, experience appears to have influenced the frequency of observation of all body areas, with the experienced observers observing all body areas significantly more frequently than the inexperienced (Figure 4). This effect was not seen with observation duration or latency to 1st fixation of the body areas.

**Figure 5:**
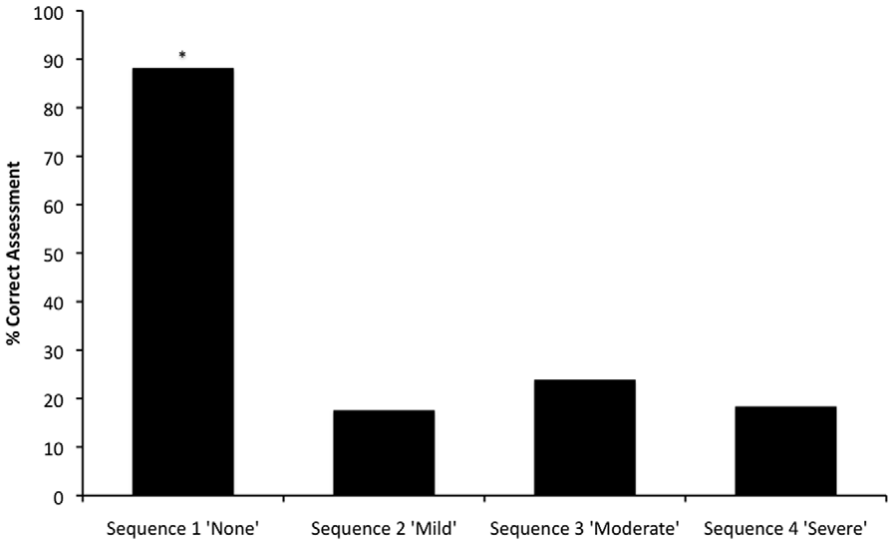
Overall percentage of correct assessments of pain for each video sequence. Video sequences is presented on the x-axis and the percentage of correct assessments is presented on the y-axis. [* = Significantly higher proportion compared to sequences 2, 3 & 4 (P<0.001)].

Overall the proportion of correct assessments of pain in each of the 4 sequences can be seen in Figure 5. The video sequence had a significant effect on the frequency with which the participants correctly assessed the pain (P<0.001). The proportion of correct assessments was significantly higher in sequence 1 (“no pain”) compared to sequences 2 (“mild pain”), 3 (“moderate pain”) and 4 (“severe pain”) (χ^2^ = 171.85, df = 3, n = 6, P<0.001 respectively). However there were no significant differences in the proportions of correct answers between the remaining sequences. There was no significant effect of either gender or experience on the frequency with which the participants correctly assessed the pain in the video sequences.

**Figure 6:**
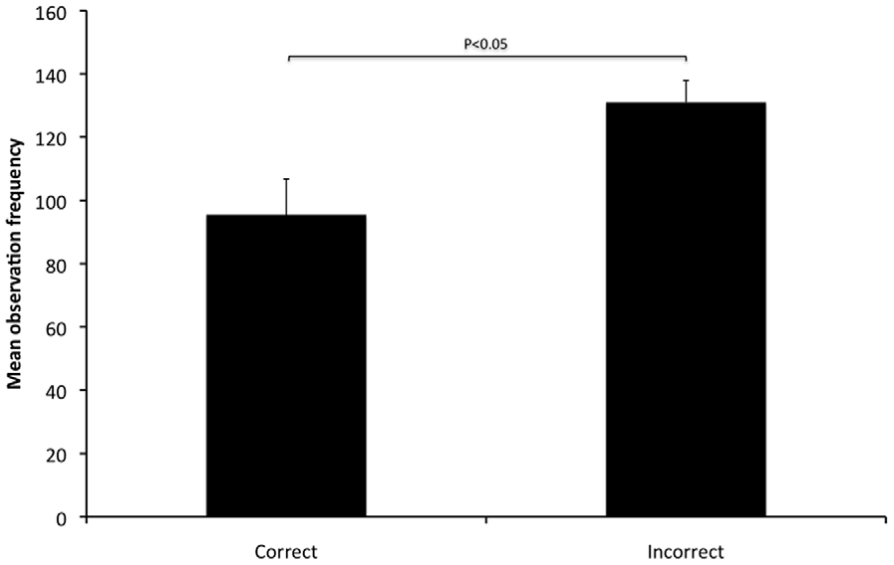
Mean observation frequency of face for correct and incorrect pain scoring. Correct and incorrect assessments for sequences 2, 3 and 4 are presented on the x-axis and the mean observation frequency of the face is presented on the y-axis (± 1SE). Significant differences are indicated on the figure.

**Figure 7:**
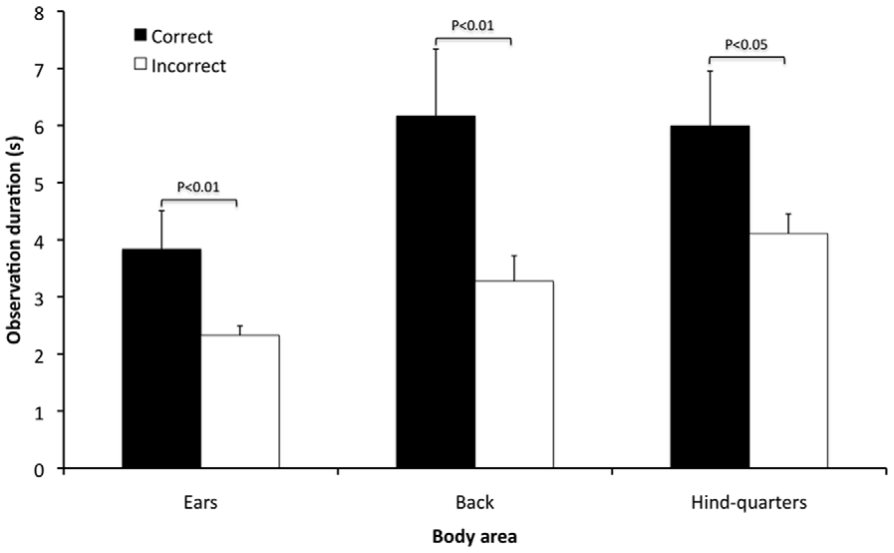
Mean observation durations of ears, back and hind-quarters for correct and incorrect pain scoring. Correct and incorrect assessments for sequences 2, 3 and 4 are presented on the x-axis and the mean observation duration (seconds) of the ears, back and hind-quarters are presented on the y-axis (± 1SE). Significant differences are indicated on the figure.

**Figure 8:**
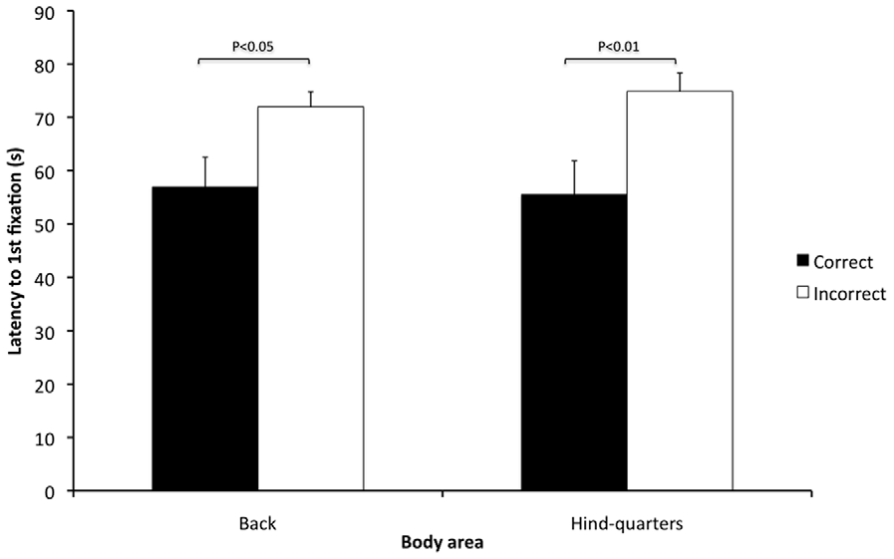
Mean latencies to 1^st^ fixation of back and hind-quarters for correct and incorrect pain scoring. Correct and incorrect assessments for sequences 2, 3 and 4 are presented on the x-axis and the mean latencies to 1^st^ fixation (seconds) of the back and hind-quarters are presented on the y-axis (± 1SE). Significant differences are indicated on the figure.

Significant biserial correlations were found between the frequency of correct assessments in the sequences of rabbits in pain (2, 3 & 4) and frequency of observation, observation duration and latency to 1^st^ fixation of the various body areas in these sequences. Observation frequency of the face was significantly higher in those who incorrectly assessed pain in the sequences compared to those who were correct (P<0.05: R_b_ = 0.173) (Figure 6). Observation duration of the ears, back and hindquarters was significantly higher in those who correctly assessed pain in the sequences compared to those who were incorrect (P<0.01: R_b_ = 0.234, P<0.01: R_b_ = 0.199, P<0.05: R_b_ = 0.165 respectively) (Figure 7). Latency to 1^st^ fixation of the back and hindquarters was significantly longer in those who incorrectly assessed pain in the sequences compared to those who were correct (P<0.05: R_b_ = 0.176, P<0.01: R_b_ = 0.188) (Figure 8). There were no other significant correlations found between the frequency of correct assessments and frequency of observation, observation duration and latency to 1^st^ fixation.

## Discussion

As far as we are aware this represents the first objective investigation of observation patterns exhibited when watching the behaviour of a non-human animal species. Although the data presented here has been corrected according to the relative size of the different body areas, this caused no discernable change in the pattern of the results over that of the uncorrected data. All observers focused first, more frequently and for longer on the face compared to the other areas of the rabbit's body irrespective of observer experience, gender or the video sequence being observed. The observers focused on the face over 3 times more frequently and longer than the remaining body areas (Figures 1 & 2). This suggests, at least with rabbits, human observers attend to the face of the animal they are observing in order to assess pain using behavioural indicators. This apparent fixation on the face exhibited by both experienced and inexperienced observers has two plausible explanations. The human tendency to focus on the face and in particular the eyes of other people when attempting to assess emotions [22] such as pain [23], [24] could be transferred to animals under circumstances that we consider to be similar. This has potential implications beyond the assessment of pain in rabbits to any observation of behaviour that is being conducted to assess how an animal feels (e.g. welfare, distress, happiness, fear) in a wide variety of contexts (e.g. farm, laboratory, rescue centre). Alternatively, the rabbits as the species being observed may account for the apparent fixation on the face. Rabbits have prominent eyes, which may simply draw the observers' attention to the eyes and face. As humans we have learnt to selectively attend to eyes [22] as they are considered an important communication device [25]. Further research is needed in order to identify why observers fixated on the face of rabbits and to determine whether the results of this study can be generalised to other situations and to other species. This research should encompass assessing observation patterns under a range of situations (e.g. during assessments of pain, distress, fear etc.) and include a range of species comprising both those with prominent and sunken eyes. If this fixation results from the human tendency to focus on the face then it should occur in a range of situations and with a variety of animal species. However, it is related to the prominence of the eyes or purely a facet of rabbits then it should only occur in species with prominent eyes or in rabbits respectively.

Observer experience had a non-significant influence on the frequency with which all the body areas were observed. Experienced observers appear to switch their focus between the body areas slightly more frequently, but still observed the different areas for a similar length of time as the inexperienced observers. This may suggest that experienced observers continually switch where they focus, as it is a better strategy for assessing behavioural indicators than observing single areas for longer periods. Alternatively experienced observers could aware that the face is not the most appropriate area to focus on and so focus elsewhere, but are subconsciously drawn back to the face after a short period. To clarify this, a more detailed assessment of how we observe animals is required.

Validated behavioural-based pain assessment schemes in rabbits [1] and other species [2]–[10] have demonstrated that that the behaviours and postures considered indicative of pain are predominately specific to the type and location of the potentially painful procedure. For example, rabbits that have undergone ovariohysterectomy exhibit pain-indicating behaviours and postures (e.g. back arching, skin twitches on the back, contraction of the abdominal muscles, belly pressing etc.) that are predominately located in the abdomen and back [1]. Therefore to recognise such changes, observers would need to focus predominantly on these two areas of the rabbit. Failure to do so will increase the chance that any behavioural and postural indicators will be missed, even if we are able to identify them. Although the observers in this study did focus some of their attention on the abdomen and to a lesser extent the back, these areas were observed infrequently, for a very short time and after a long latency from the start of the observation period. This is likely to hamper effective pain assessment in rabbits as these behavioural and postural indicators tend to be exhibited relatively infrequently and for short in duration [1]. This is emphasized by inability of the participants to score pain accurately (high proportion of incorrect scores) in the video sequences showing rabbits in pain (sequences 2–4). The importance of focusing on the back and abdomen when assessing pain following ovariohysterectomy, is further illustrated by those participants who tended to look at these areas first and for longer were also more likely to be those participants who correctly scored the pain in each sequence. Conversely, those participants that tended to focused more frequently on the face where more likely to be those participants who incorrectly scored the pain in each sequence. Although, a number of articles have suggested that our ability to effectively assess pain in animals has been limited by a lack of validated methods [1], [11]–[13], the results of this study suggest two additional explanations. Firstly, the predominant fixation on the face observed irrespective of experience increases the chance that location-specific behavioural indicators of pain may be missed. Secondly, the overall amount of time that the observers actually focused anywhere on the body of the rabbit was only just over 50% of the 1-minute video sequence (mean duration: 35 seconds [based on Figure 2]). Therefore many observers spent a relatively high proportion of the 1-minute not focusing on the rabbit and so are very likely to have missed many indicators of it's state. To clarify this further assessments of how we observe animals are required.

If a bias towards observation of the face can be demonstrated in other species, then it would suggest that people with a lower tendency to focus on the face might be better able to identify the behavioural and postural indicators of pain. This could be further investigated by assessing observation patterns and the effectiveness at assessing behaviour of human subjects that vary in their tendency to focus on the face, e.g. those with developmental disorders (e.g. autism) or those who vary in personality traits such as empathy and theory of mind. Autistic subjects attend significantly less to faces then non-autistic subjects [24], [26]. Further, the degree of empathising with other people, which is thought to be a significant factor in this variation in attending to faces, varies in different groups of individuals and professions [27]. This raises the possibility that certain professions may be more or less able to assess identify the commonly used behavioural and postural indicators of pain, i.e. a profession with high empathy, could be more likely to attend to faces, and so could miss any of these indicators when they are exhibited.

Alternatively, if we were able to identify facial expressions in animals that are associated with pain as in humans [28] then a fixation on the face of an animal may actually increase the effectiveness of pain assessment that is based upon such expressions. A recent paper by Langford et al. [29] convincingly demonstrates that mice exhibit facial expressions associated with pain. Therefore if similar expressions can be identified in other species and they are easily recognised and recorded then we could exploit our potential facial fixation to improve our ability to assess pain.

Video sequence influenced the observation frequency, duration and latency to 1st fixation of all body areas. The frequency and duration of observations for all body areas were greatest in sequence 4 (severe pain) compared to the other sequences and latency to 1st fixation of all areas of the body decreased in sequences 1 through to 4 (no pain through to severe pain). This may suggest that observers focus more closely on rabbits that are considered to be experiencing higher severities of pain. This could indicate that observers are potentially drawn to animals (either by experience or intuition) that are exhibiting deviations from normal behaviour without formal training. In such assessments observers could be using a more ‘holistic approach’ such as that proposed by Wemelsfelder & Lawerance [30], where more qualitative and descriptive indicators are used, such as ‘calm’, ‘anxious’, ‘timid’ and ‘confident’ etc. However, if this is the case, it did not improve the ability of the participants to correctly score the rabbit's pain as the proportion of correct answers was equally low (18–24%) in sequences 2 to 4 (mild to severe pain) compared to sequence 1 (no pain: 88%) irrespective of gender or experience. However further studies are needed using more than one video sequence per category of pain severity to confirm this preliminary finding. If observers are ‘drawn’ to animals in pain then it would suggest that the assessment of pain could easily be improved through training observers in not only which behaviours, facial expressions and postures to record but also what areas of the body to focus upon to effectively observe these indicators. Training observers on what behaviours to look for has been shown to improve their ability to assess animal pain [11].

We would suggest that this potential bias in our attention to specific body areas in rabbits and potentially other species could be of direct relevance to any assessment of how an animal feels that uses behaviour. Effective observation of behaviour for whatever the ultimate purpose depends equally on knowing which behaviours to look for and attending to the correct area or areas of the body to observe these behaviours.

## Materials and Methods

### Video sequences

Four 1-minute video sequences of singly housed free-moving New Zealand white rabbits (*Oryctolagus cuniculi*) were shown to the participants. Each sequence showed a different individual in their home pen (1 m×2 m) that was adjacent to other rabbits and contained sawdust bedding, a plastic cat litter tray, a clean cardboard tube, a pine rabbit chew block and ad libitum access to food and water. There were no obvious discernable physical differences between the rabbits in each sequence.

Each sequence showed a rabbit that had undergone routine ovariohysterectomy and was considered to be experiencing a different severity of pain (Table 1) according to a structured behaviour-based assessment carried by an experienced research worker using behavioural and postural indicators of rabbit pain (Table 2).

**Table 1:** Pain severity classification and description of each of the video sequences observed.

Sequence	Severity	Description
1	Normal	Exhibiting no pain related behaviour or postures
2	Mild	Exhibiting less than 2 pain related behaviour or postures
3	Moderate	Exhibiting between 3–5 pain related behaviour or postures
4	Severe	Exhibiting greater than 6 pain related behaviour or postures

**Table 2:** Ethogram of pain behaviour used to determine the pain severity of each sequence.

Behaviour	Description
Twitch	Rapid movement of fur on back
Flinch	Body jerks upwards for no apparent reason.
Wince	Rapid movement of the backwards in a rocking motion accompanied by eye closing and swallowing action
Stagger	Partial loss of balance
Fall	Complete loss of balance when moving
Press	Abdomen pushed towards floor, usually before walking
Arch	Full arching of the back upwards
Writhe	Contraction of the oblique flank muscles
Shuffle	Walking at a very slow pace
Quiver	Slow rhythmic side-to-side movement

Behavioural and postural indicators of rabbit pain following ovariohysterectomy [1].

### Ethical statement

The four sequences shown to the participants were taken from an extensive historical video archive and were recorded during an unrelated analgesic efficacy study in 2006 that employed a strict ‘rescue’ analgesia policy. If any animal was deemed to be in greater than mild pain (assessed by independent veterinarian), then buprenorphine (0.01 mg/kg iv) was immediately administered and the animal was removed from the study. The analgesic efficacy study was carried out under project and personal licences approved by the Secretary of State for the Home Office, under the United Kingdom's 1986 Animals (Scientific Procedures) Act and the local ethical review committee at Newcastle University. Consequently no animals underwent surgery or were directly used in order to record data for the purposes of this vision tracking study. Verbal informed consent was gained from each participant prior to taking part in this study. Written consent was deemed as unnecessary as no personal details of the participants were recorded. This study did not require institutional review board approval in order for it to be carried out.

### Equipment

Data was recorded using Gazetracker (Eye Response Technologies, Charlottesville, USA) and 50 Hz Video Eyetracker Toolbox version 3.21 (Cambridge Research Systems, Rochester, UK). The Eyetracker toolbox was running on a Dell Inspiron PC (Dell, UK) with the video sequences being shown via a Samsung 17-inch LCD monitor (720N: Samsung, China). For reasons of brevity, technical details of how the data was recorded and processed by the Gazetracker hardware and Eyetracker toolbox software will not be presented in this manuscript. A full explanation can be found in Collewijn [31] or on the Cambridge Research Systems website (http://www.crsltd.com).

### Participant selection

A total of 126 observers participated in this study (equal numbers of male & female). They were recruited and tested in 2008 at one of three venues: Institute of Animal Technology Annual Congress (n = 11), Bristol University Veterinary School (n = 70) and Newcastle University Comparative Biology Centre (n = 45). The observers were from diverse backgrounds and included veterinary surgeons, veterinary nurses, research scientists, animal technicians, psychology students and non-animal related occupations. Observers were classed into two categories according to their prior experience with rabbits. The experienced observers (n = 61) included those people who cared for rabbits in a veterinary setting, those who kept them as pets and those who worked with them in a research setting. The inexperienced observers (n = 65) had no previous experience of caring for, working with or keeping rabbits.

### Procedure

**Figure 9:**
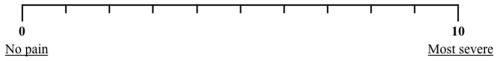
The line used for the visual analogue scoring of pain in each sequence. For each sequence the participants were asked to place a mark on the line at the point they score the pain the rabbit was experiencing by placing a mark on the line at the point they felt corresponded to the pain experienced by the rabbit. They were told that 0 represented no pain and 10 represented the most severe pain they could imagine.

Each observer initially completed a short questionnaire detailing his or her gender, occupation and rabbit-related experience (no experience, work with them, and/or keep them as pets). On completion of the questionnaire the equipment was calibrated for each individual observer. They then viewed the four 1-minute sequences in a randomised order with a 1-minute break between sequences. The participants were asked to assess the pain exhibited by the rabbit in each sequence using their intuition (no training in pain assessment was provided) by means of visual analogue scoring. On completion of each sequence the participants were asked to place a mark on a 10 cm line at the point at which they felt corresponded to the pain experienced by the rabbit in that sequence (Figure 9). They were told that on this line 0 represented no pain and 10 represented the most severe pain they could imagine. The observers were unaware of the pain severity category of each of the sequences they were shown.

### Body area scoring

**Figure 10:**
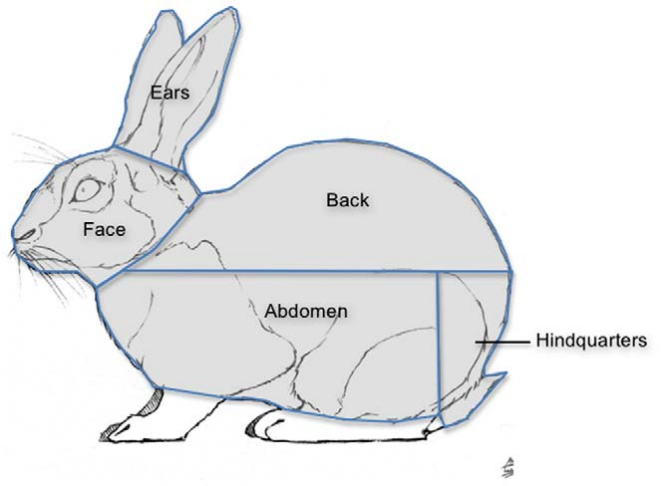
The sub-division of the body into the five areas for scoring during observations. The body of the rabbit in each video sequence was sub-divided into 5 distinct areas (ears, face, back, abdomen and hindquarters) that were automatically scored during participant observations.

In order to score which areas of the body the observers focused upon while watching each sequence, the rabbits' body was divide into 5 areas; face (face, head and neck, but excluding the ears), ears (ears only), abdomen, back and hindquarters (Figure 10) using Video Eyetracker Toolbox. In every frame of each sequence the 5 above areas of the body were visually defined and highlighted irrespective of the orientation of the rabbit. This enables the toolbox to recognise when a participant's gaze enters these pre-defined areas during video playback.

### Analysis

Data on the frequency and duration of observation and the latency to 1st fixation of each area of the rabbit's body and the size (in Pixels) of each body area were recorded and exported for further analysis from the Eyetracker toolbox (Table 3). This data was imported into Excel (Microsoft Inc, Seattle, USA) for sorting and then exported to SPSS (SPSS Inc., Chicago, USA) for all statistical analysis. Differences were considered to be statistically significant at p≤0.05.

**Table 3:** Description of the measures exported from Eyetracker toolbox for further analysis.

Measure	Description
Observation frequency	The frequency that each of the 5 areas of the rabbit's body was observed in each 1-minute video sequence.
Observation duration	The duration (in seconds) that each of the 5 areas of the rabbit's body was observed in each 1-minute video sequence.
Latency to 1^st^ fixation	The latency (in seconds) before each of the 5 areas of the rabbit's body was observed in each 1-minute video sequence.

In order to ensure that any significant differences found between the various body areas were not related to differences in their relative size, a simple mathematical correction was applied to the data prior to analysis. This involved calculating the relative proportion of each area compared the largest size (the ears) (Table 4). These proportions were then used to correct the frequency, duration and latency to 1st fixation data by multiplying each value by the respective proportion.

**Table 4:** Overall surface area (pixels) and relative proportions of the body areas.

Body area	Area (Pixels)	Proportion
Ears	10146	1
Face	6900	1.47
Back	5304	1.91
Abdomen	3840	2.64
Hindquarters	5145	1.97

Relative proportions of the face, back, abdomen and hindquarters compared to the ears (largest area) are shown. These proportions were used in the correction of the frequency, duration and latency to 1^st^ fixation data (see methods for details).

The data imported from the eye-tracking software was normally distributed with homogenous variance, so parametric analysis was used. The data was analysed using a Repeated Measures General Linear Model (GLM). The within-subjects factors were body area (5 levels: face, ears, back, abdomen and hindquarters) and sequence (4 levels: 1, 2, 3 and 4). The between-subjects factors were observer experience (2 levels: non experience and experienced) and gender (2 levels: male and female). Significant mean differences between the within-subjects factors were tested post-hoc using paired student t-tests with a Bonferroni correction multiple comparisons.

The data referring to correct/incorrect assessment of pain in each of the four sequences was not normally distributed with heterogeneous variance, so non-parametric analysis was used. Mann-Whitney U tests were used to compare the frequency of correct answers by gender (2 levels: male and female) and experience (2 levels: non experience and experienced). A Freidman test was used to compare the frequency of correct answers between the 4 video sequences (4 levels: 1, 2, 3 and 4). Significant mean differences between the video sequences were tested post-hoc using Wilcoxon tests with a Bonferroni correction multiple comparisons.

The relationship between measure of observation pattern (observation frequency, observation duration and latency to 1^st^ fixation) and correct/incorrect assessment of pain (2 levels) was tested with Biserial correlations for sequences 2, 3 and 4. Sequence 1 was not included in the analysis as it referred to rabbits in a ‘no pain’ state.
